# Supplementary figures and images for: Exploring the relationships between eco-anxiety, eco-guilt, eco-grief, and pro-environmental behavior in the Dutch and German population: A cross-sectional study
Source: PLoS One. 2026 May 19;21(5):e0349585. doi: 10.1371/journal.pone.0349585 (PMC13186368; doi:10.1371/journal.pone.0349585)

**S2 Appendix**

**S2 Fig. Pathways moderated mediation model.**


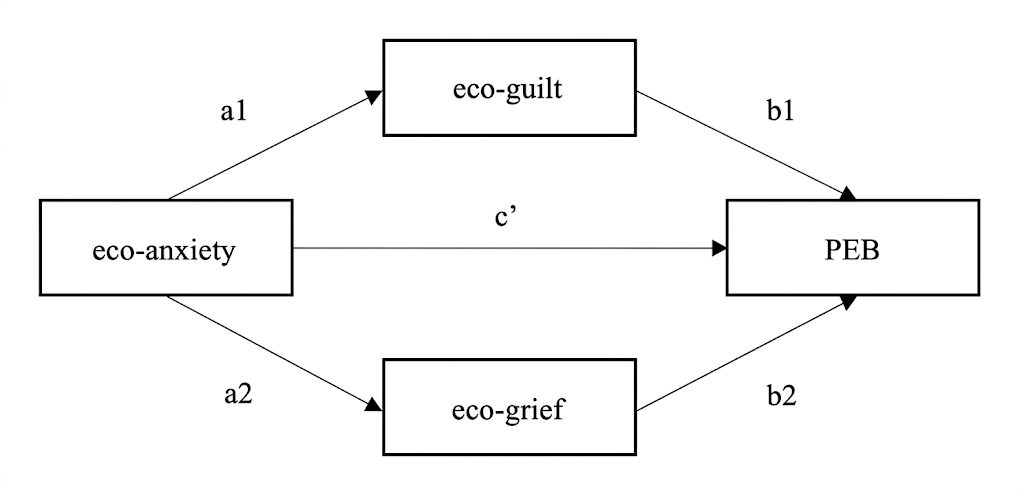

Supplement: S1 Fig — (DOCX) [file pone.0349585.s002.docx]

**S3 Appendix**

**S3 Fig. Boxplots of gender and eco-anxiety, eco-guilt, eco-grief, and PEB.**

*
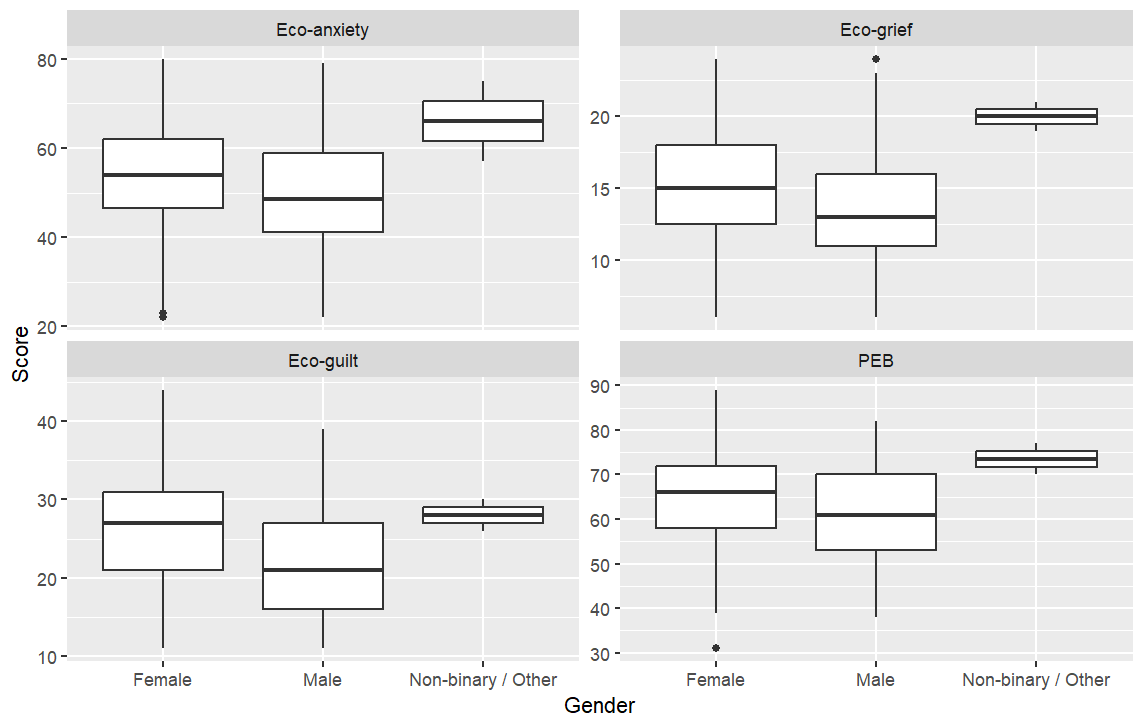
*

Supplement: S2 Fig — (DOCX) [file pone.0349585.s003.docx]

**S4 Appendix**

**S4 Fig. Boxplots of nationality and eco-anxiety, eco-guilt, eco-grief, and PEB.**

*
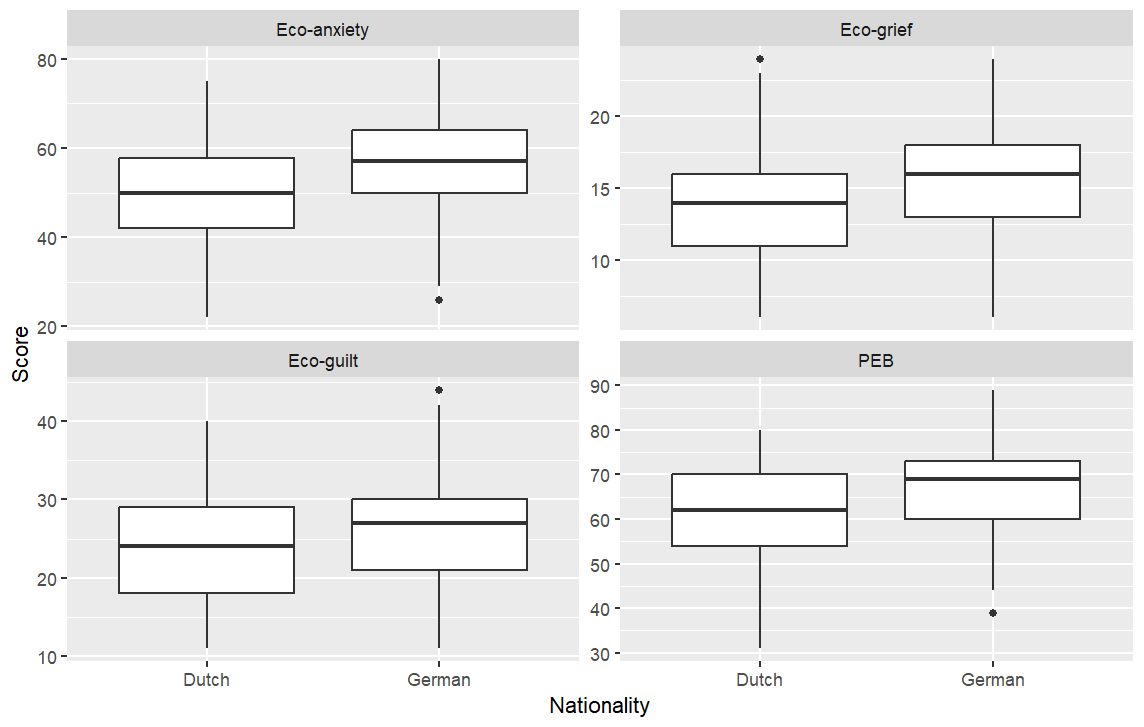
*

Supplement: S3 Fig — (DOCX) [file pone.0349585.s004.docx]

**S5 Appendix**

**S5 Fig. Boxplots of proximity to water and eco-anxiety, eco-guilt, eco-grief, and PEB.**

*
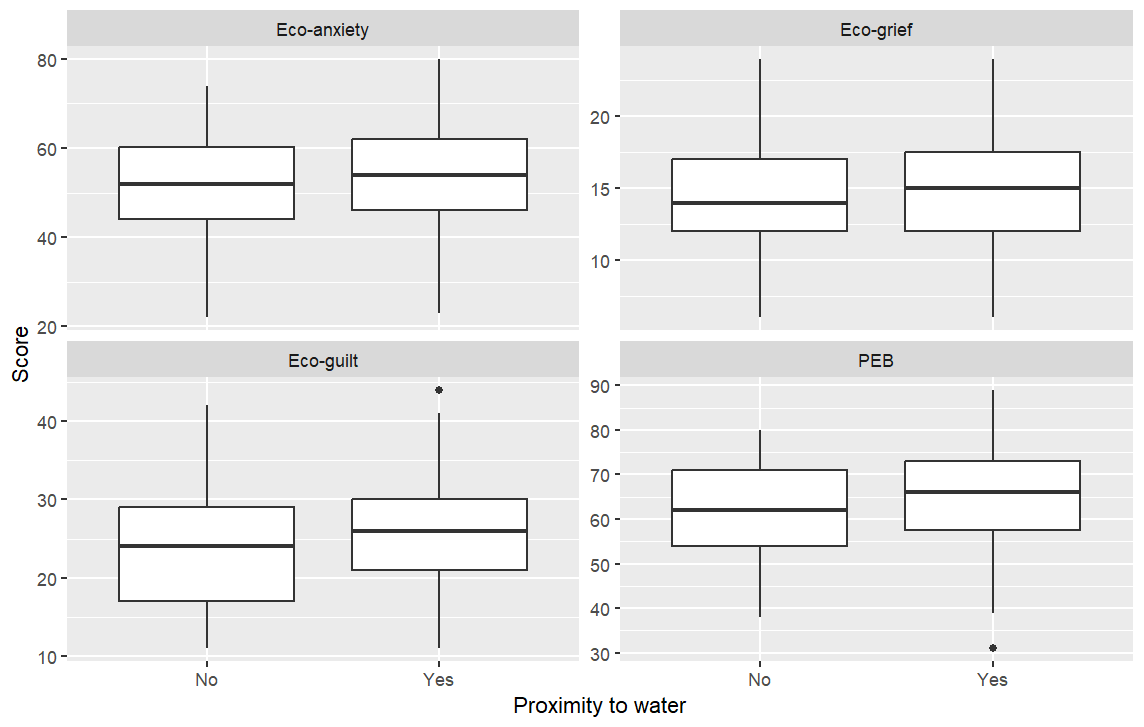
*

Supplement: S4 Fig — (DOCX) [file pone.0349585.s005.docx]
